# Investigating the Effects of ONC206 Alone and in Combination with Cisplatin on Ovarian Cancer Cell Models

**DOI:** 10.3390/cimb47060451

**Published:** 2025-06-12

**Authors:** Sara Mikhael, Rona Fayyad, Leen Abi Harfouch, Varun Vijay Prabhu, Hisham F. Bahmad, Wassim Abou-Kheir, Georges Daoud

**Affiliations:** 1 Department of Anatomy, Cell Biology and Physiological Sciences, Faculty of Medicine, American University of Beirut, Beirut 1107 2020, Lebanon; srm.mikhael@gmail.com (S.M.); rrf10@mail.aub.edu (R.F.); lwa13@mail.aub.edu (L.A.H.); wa12@aub.edu.lb (W.A.-K.); gd12@aub.edu.lb (G.D.); 2 Chimerix, Inc., Durham, NC 27713, USA; vprabhu@chimerix.com; 3 Department of Pathology and Laboratory Medicine, University of Miami Miller School of Medicine, Miami, FL 33136, USA

**Keywords:** ovarian cancer, chemotherapy, imipridones, 3D cell culture, cancer stem cells, synergy analysis

## Abstract

Ovarian cancer (OC) is the most lethal gynecologic malignancy worldwide, with high rates of disease relapse posing a significant therapeutic challenge. Consequently, there is an urgent need to develop novel treatments for OC. This study aims to evaluate the effects of the novel imipridone, ONC206, both as a monotherapy and in combination with the standard of care chemotherapy drug, cisplatin (CDDP), on human OC cell lines. In order to study the effect of ONC206 and CDDP on ovarian cancer, two cell lines, OVCAR-420 and SKOV-3, were used in this study. Cell proliferation was assessed using MTT assay while cell viability was evaluated using the trypan blue exclusion assay. Cell migration was examined using the wound healing assay. To investigate the effects of both treatments, alone or in combination on the stem-cell-like population of OC cells, the sphere-forming assay was employed. Our results revealed that ONC206, alone or in combination with CDDP, exerts a potent anti-proliferative effect on both OVCAR-420 and SKOV-3 cells, as shown in the MTT and trypan blue exclusion assays. Interestingly, a synergistic effect was observed when ONC206 was combined with CDDP, enhancing the overall anti-cancer efficacy. Additionally, ONC206 alone or in combination with CDDP inhibited the migratory ability of the ovarian cancer cells. Furthermore, the activity of ovarian cancer stem cells was inhibited when cells were treated with ONC206 alone or in combination with CDDP, as shown in the significant decrease in both the size and the sphere-forming ability of ovarian cancer stem cells in the 3D culture model. Our results highly suggest the potential of imipridones as a new class of therapeutics in ovarian cancer management. Among these, ONC206 shows nanomolar potency, highlighting its potential as a standalone therapy or in combination with existing treatment regimens.

## 1. Introduction

Ovarian cancer (OC) represents a significant health burden globally, being the most lethal gynecological malignancy and ranking among the top five cancers causing mortality in women worldwide [1]. Ovarian cancer (OC) diagnosis primarily depends on the pathological analysis of a biopsy. The disease is characterized by significant genetic variability and marked phenotypic heterogeneity [2]. According to the 5th edition of the World Health Organization (WHO) guidelines, ovarian neoplasms are classified into two broad categories: epithelial and non-epithelial tumors. Epithelial tumors account for approximately 90% of all cases and include high-grade serous carcinoma (HGSC), low-grade serous carcinoma (LGSC), mucinous carcinoma (MC), endometrioid carcinoma (EC), and clear cell carcinoma (CCC) [3]. Each subtype exhibits distinct features regarding origin, patterns of metastasis, treatment response, and clinical outcome [4]. The remaining 10% of ovarian neoplasms consist of non-epithelial tumors, which include mesenchymal (stromal) tumors, mixed epithelial and mesenchymal tumors, germ cell tumors, sex cord-stromal tumors, miscellaneous tumors, tumor-like lesions, and secondary tumors [3]. Prognosis heavily relies on the disease type and stage at diagnosis as well as the response to therapy [5]. Early-stage OC (Stage I and II) shows a relatively favorable 5-year overall survival rate of around 90% and 70%, respectively. However, when the disease is diagnosed at a late stage (Stages III and IV), where it has spread beyond the pelvis, survival rates dramatically decline to approximately 20% [6].

Accurate classification and staging are pivotal for devising effective treatment regimens. Current treatment options encompass surgery, chemotherapy, radiation therapy, and targeted therapies [7]. Surgical intervention is most beneficial in early-stage disease for tumor debulking [7,8]. However, chemotherapy remains the cornerstone for advanced OC with taxane–platinum combinations being the most widely used regimen [8,9]. Despite their efficacy, these treatments are accompanied by significant drawbacks, such as severe systemic toxicity, off-target effects, and the eventual development of chemoresistance, which limits their long-term effectiveness and contributes to poor overall survival rates [6,10]. A major factor contributing to chemoresistance in OC is the presence of cancer stem cells (CSCs), a subpopulation of cells within the tumor that possess self-renewal properties and are thought to drive tumor initiation, progression, and recurrence. CSCs are notoriously resistant to conventional chemotherapy due to their ability to remain in a quiescent state, up-regulate drug efflux transporters, and repair DNA damage more efficiently than non-stem cancer cells. As a result, CSCs are able to survive treatment and contribute to disease relapse and metastasis [11,12]. CSCs have been detected in both hematologic cancers and numerous solid tumors [12]. Actually, the first experimental evidence supporting the presence of ovarian CSCs emerged in 2005, when Bapat and colleagues [13] isolated a tumorigenic clone from the malignant ascites of an ovarian cancer patient using a multilayer spheroid culture system. However, despite enormous advancements in cancer treatment, OC has seen limited progress compared to other malignancies. Reducing the risk of relapse and enhancing treatment response in advanced OC patients remain critical clinical hurdles [7].

Given the limited efficacy of current therapies, there is a pressing need for novel therapeutic approaches that can overcome chemoresistance and improve outcomes for OC patients. Dordaviprone/ONC201 and its potent analog ONC206 belong to the imipridone family and represent innovative small molecules with distinctive chemical structures. They act as both an antagonist of dopamine receptor D2/3 (DRD2/3) and an agonist of mitochondrial caseinolytic protease P (ClpP). Imipridones, including ONC206, induce the integrated stress response (ISR) in tumor cells and promote the activity of TNF-related apoptosis-inducing ligand (TRAIL), a cytokine known for its selective targeting and elimination of tumor cells [14]. ONC206 is presently undergoing phase I clinical trials for adult and pediatric patients with primary brain tumors. ONC206 has shown efficacy in mouse models of various cancers, including high grade glioma, hepatocellular carcinoma, endometrial, and ovarian cancer [15,16,17,18,19]. Moreover, Tucker et al. compared the effects of ONC206 with ONC201 in OC models [19]. In this study, they showed that ONC206 exhibited a ten-fold increase in potency when compared to ONC201 in reducing cell proliferation in SKOV-3 and OVCAR-5 cell lines. Furthermore, they revealed significant suppression of cellular adhesion and invasion in vitro, as well as inhibition of proliferation through the induction of G1 cell cycle arrest and apoptosis. However, there is still a notable gap in the literature regarding the effects of ONC206 in OC either alone or in combination with the platinum treatment regimen.

Therefore, in this study, we aimed to investigate the impact of ONC206 on OC cells and compare its efficacy with that of cisplatin. In particular, we sought to explore its effects on CSCs using a 3D cell culture system. Additionally, we explored the potential synergistic interaction between ONC206 and cisplatin. While cisplatin remains a mainstay in OC treatment, its non-specific toxicity and the development of resistance limit its clinical efficacy [20]. Therefore, combining cisplatin with a novel agent like ONC206 which operates through distinct mechanisms may offer a complementary approach.

## 2. Materials and Methods

### 2.1. Cell Culture

The human ovarian cancer cell lines OVCAR-420 and SKOV-3 were kindly provided by Dr. Wassim Abou Kheir (American University of Beirut, Lebanon). Both cell lines were cultured and maintained in RPMI-1640 (Sigma-Aldrich, Inc., St. Louis, MO 63178, USA) with 10% heat-inactivated fetal bovine serum (FBS; Sigma-Aldrich, Inc., St. Louis, MO 63178, USA), 1% penicillin–streptomycin (PS) (Sigma-Aldrich, Inc., St. Louis, MO 63178, USA) and Plasmocin^TM^ prophylactic (InvivoGen, Toulouse, France). The cells were maintained in a humidified atmosphere at 37 °C with 5% CO_2_.

### 2.2. Drugs

Cisplatin (CDDP; cis-diammineplatinum (II) dichloride) (Santa Cruz Biotechnology, Inc., 69115 Heidelberg, Germany: CAS no: 15663-27-1) was reconstituted in 1.8% NaCl to reach a concentration of 0.5 mg/mL (1.7 mM) and stored at −20 °C.

ONC206 was provided by Oncoceutics Inc. (Philadelphia, PA, USA) and reconstituted in dimethyl sulfoxide (DMSO; cat. No. D2650; Sigma-Aldrich, Inc., St. Louis, MO 63178, USA), per manufacturer’s instructions, at 20 mM and stored at −20 °C.

### 2.3. MTT

The MTT (3-(4,5-dimethylthiazol-2-yl)-2,5-diphenyltetrazolium bromide) assay was performed to assess the effect of CDDP or ONC206, either alone or in combination, on the proliferation of the SKOV-3 and OVCAR-420 cell lines. Briefly, cells were plated and grown in a 96-well plate at a concentration of 10, 8, and 6 × 10^3^ cells/well for 24, 48, and 72 h, respectively. Cells were treated with varying concentrations of cisplatin (CDDP) (0–100 µM) and ONC206 (0–10 µM), either alone or in combination, for the indicated time points.

### 2.4. Trypan Blue Exclusion Method

Trypan blue assay was performed to assess the effects of CDDP and ONC206, alone or in combination, on the viability of the SKOV-3 and OVCAR-420 cell lines. A total of 6 × 10^4^ cells/well were seeded in a 24-well plate for 24 h before treatment with different concentrations of CDDP (0–20 µM) and ONC206 (0–1 µM) for 48 h. Cells were harvested, counted, and results were expressed as the percentage growth relative to untreated cells.

### 2.5. Synergy Analysis

Synergy analysis was performed using CompuSyn software (version 1.0.1) according to Chou and Talalay’s method [21]. These experiments were conducted in triplicate, and the Fraction affected (Fa) values, representing the fraction of cells inhibited by the drug, were determined. Synergism, additivity, or antagonism in the different combinations were calculated using the combination index (CI), where CI < 1 indicated synergism, CI = 1 indicated an additive effect, and CI > 1 indicated antagonism.

### 2.6. Wound Healing Assay

To assess the effect of combining ONC206 and standard chemotherapy on the cell migration for both ovarian cancer cell lines, a wound healing assay was used. OVCAR-420 and SKOV-3 cells (180 × 10^3^ cells/well) were seeded in 24-well culture plates and incubated at 37 °C and 5% CO_2_ until they were 90–100% confluent. Cells were then treated with 0.01 mg/mL of Mitomycin C (Sigma-Aldrich, Inc., St. Louis, MO 63178, USA) for 5 min to inhibit cellular proliferation. A mid scratch was performed on each well using a sterile 200 μL pipet. Then, phosphate-buffer saline (PBS) was used to wash the monolayer of cells twice to remove any detached cells and debris. Each condition was performed in duplicate, and cells were either treated with vehicles alone or with the respective drug. Photographs of the wounds were taken using inverted light microscopy at the following time points: 0, 6, 12 h. The distance between the scratches was measured using ZEN Microscope Software (Zen 2.3). The equipment are from ThermoFisher SCIENTIFIC (Waltham, MA 02451, USA).

### 2.7. Three-Dimensional Culture and Sphere-Formation Assay

The 3D sphere-formation assay was performed to investigate the effect of CDDP and ONC206, either alone or in combination, to target the self-renewing OCSCs in SKOV-3 and OVCAR-420 cell lines. A total of 1500 cells were seeded in a cold Matrigel^TM^/serum free RPMI (1:1 dilution). A total of 20 μL and 10 μL of the OVCAR 420 and SKOV-3 mixture, respectively, was carefully plated around the rim of each well of a pre-heated 96-well plate. The mixture was allowed to solidify at 37 °C in a humified incubator for 20 min. Afterwards, 100 μL of complete media containing 5% FBS was added to OVCAR-420 cells with different drug concentrations of CDDP (0.5 and 2.5 µM) and/or ONC206 (0.025 and 0.1 µM). SKOV-3 cells were treated with CDDP (0.1 and 1 µM) and/or ONC206 (0.01 and 0.05 µM). Culture media containing the respective treatments were replenished every other day as per the original seeding. After 7 and 10 days for OVCAR-420 and SKOV-3, respectively, the spheres were counted and bright field images were acquired using Axiovert microscope from Zeiss at 10× magnification. Images were analyzed by Carl Zeiss Zen 2012 image software to determine sphere sizes. The sphere-formation unit (SFU) was calculated as follows:

$$\mathrm{SFU}=\frac{number\;of\;spheres\;formed\;}{number\;of\;cells\;originally\;plated}\;\times \;100$$

Results were represented as the percentage of the SFU and size of the treated spheres compared to the control ones. The data were derived from the mean of duplicate wells of the three independent experiments.

### 2.8. Statistical Analysis

Statistical analysis was performed using GraphPad prism (version 9.5.1, GraphPad Software Inc., La Jolla, CA, USA) and data were presented as mean ± standard error of the mean (SEM). A two-way ANOVA test was used to analyze the results of the MTT, whereas a one-way ANOVA test was performed for the analysis of the trypan blue assay, wound healing assays, and sphere-formation assay. Statistical significance was reported at *p*-values of *p* < 0.05 (*), *p* < 0.01 (**), and *p* < 0.001 (***).

## 3. Results

### 3.1. Increased Expression of DRD2 and CLPP in Ovarian Tumors

**Figure 1:**
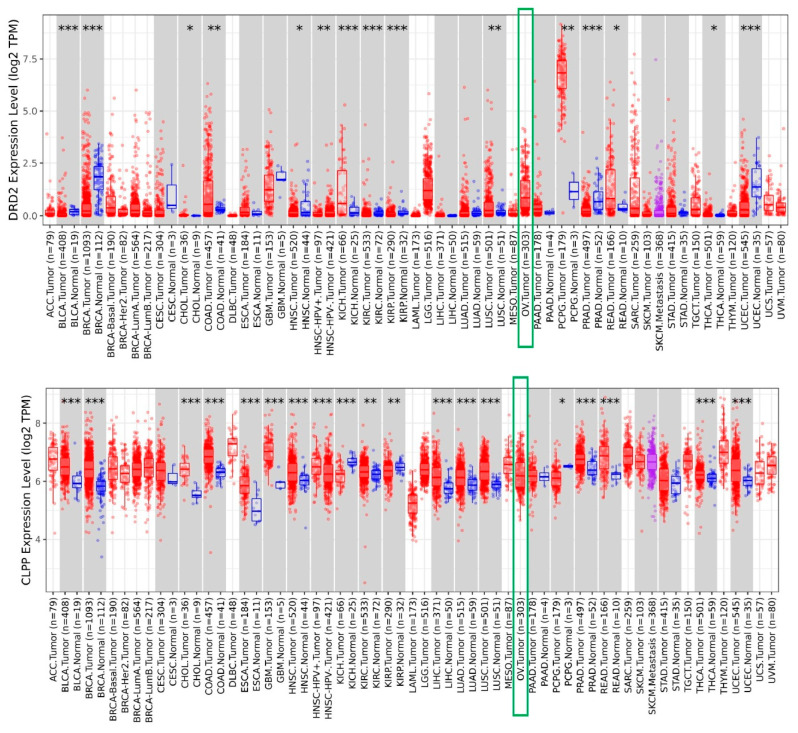
Increased expression of *DRD2* and *CLPP* in ovarian tumors. The graph demonstrates the differential expression of *DRD2* and *CLPP* across TCGA tumors. Distributions of gene expression levels are displayed using box plots. The statistical significance computed by the Wilcoxon test is annotated by the number of stars (*: *p*-value < 0.05; **: *p*-value < 0.01; ***: *p*-value < 0.001) [22]. Abbreviations: ACC: Adrenocortical Carcinoma; BLCA: Bladder Urothelial Carcinoma; BRCA: Breast Invasive Carcinoma; CESC: Cervical and Endocervical Cancer; CHOL: Cholangiocarcinoma; COAD: Colon Adenocarcinoma; DLBC: Diffuse Large B-cell Lymphoma; ESCA: Esophageal Carcinoma; GBM: Glioblastoma Multiforme; HNSC: Head and Neck Cancer; KICH: Kidney Chromophobe; KIRC: Kidney Renal Clear Cell Carcinoma; KIRP: Kidney Renal Papillary Cell Carcinoma; LAML: Acute Myeloid Leukemia; LGG: Lower Grade Glioma; LIHC: Liver Hepatocellular Carcinoma; LUAD: Lung Adenocarcinoma; LUSC: Lung Squamous Cell Carcinoma; MESO: Mesothelioma; OV: Ovarian Serous Cystadenocarcinoma; PAAD: Pancreatic Adenocarcinoma; PCPG: Pheochromocytoma and Paraganglioma; PRAD: Prostate Adenocarcinoma; READ: Rectum Adenocarcinoma; SARC: Sarcoma; SKCM: Skin Cutaneous Melanoma; STAD: Stomach Adenocarcinoma; TGCT: Testicular Germ Cell Tumor; THCA: Thyroid Carcinoma; THYM: Thymoma; UCEC: Uterine Corpus Endometrial Carcinoma; UCS: Uterine Carcinosarcoma; UVM: Uveal Melanoma.

Dordaviprone/ONC201 and its potent analog ONC206 act as both an antagonist of dopamine receptor D2/3 (DRD2/3) and an agonist of mitochondrial caseinolytic protease P (ClpP). To assess *DRD2* and *CLPP* expression in human cancers, we verified the different expression for both genes across TCGA tumors by the TIMER database. TIMER 2.0 (http://timer.cistrome.org/), a comprehensive online tool, was applied to explore the tumor differential expression for *DRD2* and *CLPP* among all The Cancer Genome Atlas (TCGA) tumors [22]. As shown in Figure 1, both genes were up-regulated in ovarian serous carcinomas (green boxes).

### 3.2. ONC206 and CDDP Decrease the Proliferation of Ovarian Cancer Cells

**Figure 2:**
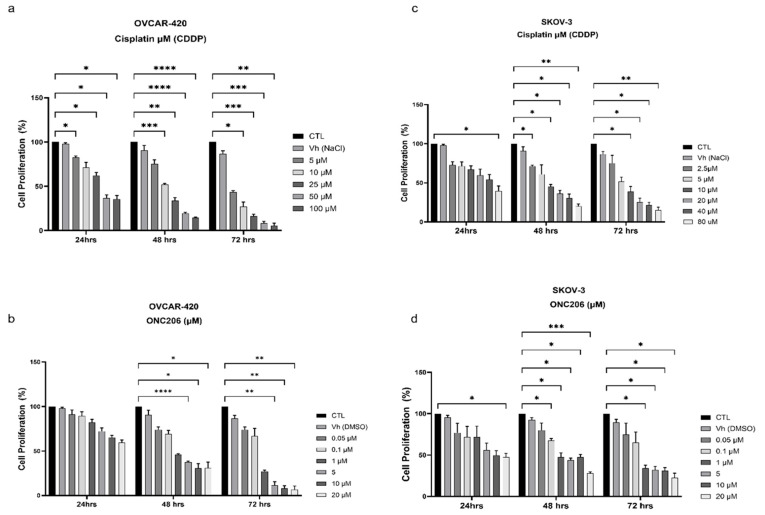
ONC206 and CDDP decrease the proliferation of OVCAR-420 and SKOV-3. The effect of increasing concentrations of ONC206 and CDDP on the proliferation of OVCAR-420 (**a**,**b**) and SKOV-3 (**c**,**d**) cells using the MTT assay was determined in triplicate at 24, 48, and 72 h. Results are expressed as a percentage of the proliferation of the treated group compared to the control at every time point. Data represent the mean ± SEM of three independent experiments and are analyzed using two-way ANOVA (* *p* < 0.05; ** *p* < 0.01; *** *p* < 0.001; **** *p* < 0.0001).

To assess the individual effects of CDDP and ONC206 on ovarian cancer cells, OVCAR-420 cells and SKOV-3 were treated with increasing concentrations of CDDP (2.5 μM to 100 μM) and ONC206 (0.05 μM to 20 μM) for 24, 48, and 72 h. The MTT assay was used to measure metabolic activity, which indirectly reflects cellular proliferation. Both drugs induced a significant decrease in cell proliferation, in a time- and dose-dependent manner (Figure 2). The half-maximal inhibitory concentration (IC_50_) for each drug was calculated at each time point (Table 1). Cells treated with the vehicle (NaCl or DMSO) showed no effect on cellular proliferation.

### 3.3. ONC206 Reduces the Proliferation of Ovarian Cancer Cells Alone and in Combination with CDDP

**Figure 3:**
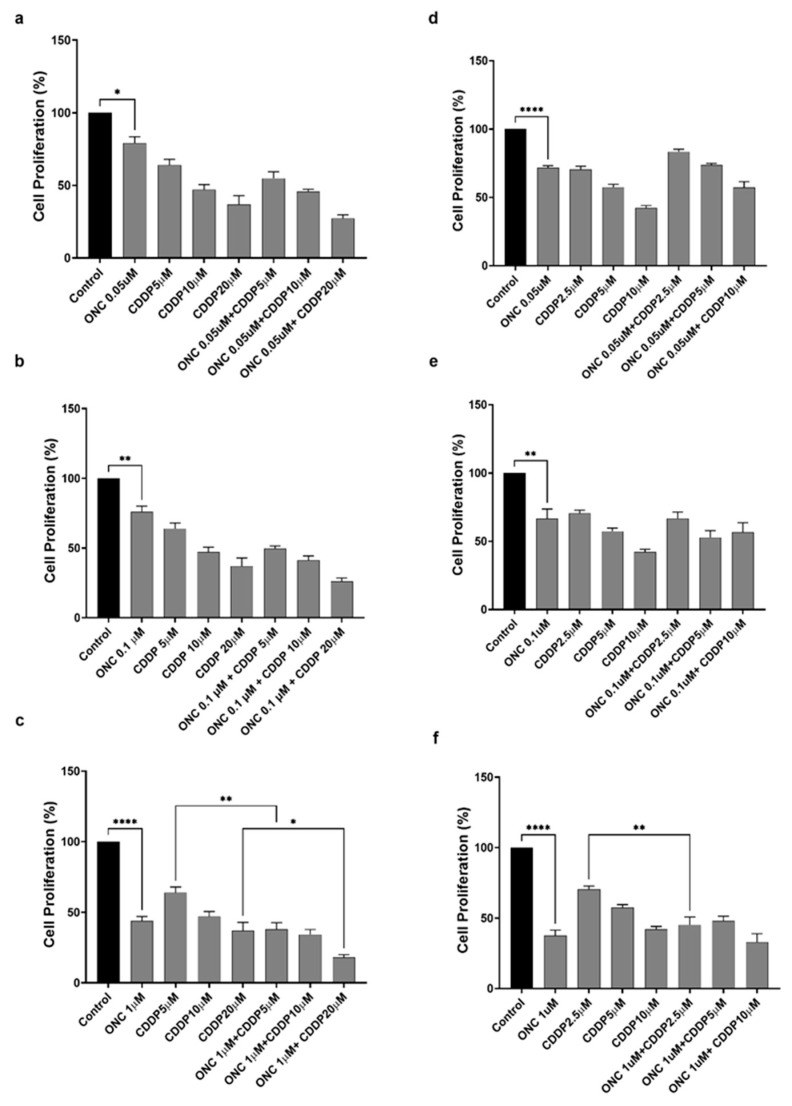
ONC206 reduces cell proliferation of OVCAR-420 and SKOV-3 alone and in combination with CDDP. The effect of different concentrations of ONC206 and CDDP combinations on the proliferation of OC cells using MTT assay was determined at 48 h. (**a**–**c**) OVCAR-420 and (**d**–**f**) SKOV-3. Results are expressed as a percentage of the proliferation of the treated group compared to the control at every time point. Data represent the mean ± SEM of three independent experiments and are analyzed using two-way ANOVA (* *p* < 0.05; ** *p* < 0.01; **** *p* < 0.0001).

To assess the effects of CDDP and ONC206, both alone or in combination, on the proliferation of ovarian cancer cells, SKOV-3 and OVCAR-420 were treated for 48 h with increasing concentrations of ONC206 (0.05, 0.1, and 1 µM) and CDDP (5, 10, and 20 µM for OVCAR-420 or 2.5, 5, and 10 µM for SKOV-3). Using the MTT assay, the results showed that ONC206 and CDDP significantly reduced metabolic activity in both cell lines in a dose-dependent manner. ONC206 alone induced a 20–30% reduction at concentrations of 0.05 and 0.1 µM, and an approximately 40–50% reduction at 1 µM (Figure 3). CDDP alone caused a 20–50% reduction, depending on the concentration. Interestingly, when cells were co-treated with 1 µM ONC206, and any tested CDDP concentration, this yielded a statistically significant synergistic reduction, with a 50–80% decrease in metabolic activity compared to CDDP alone (Figure 3c,f). These findings are reflective of an anti-proliferative effect, warranting further investigation using direct proliferation assays.

### 3.4. ONC206 Decreases the Cellular Viability of Ovarian Cancer Cells Alone and in Combination with CDDP

**Figure 4:**
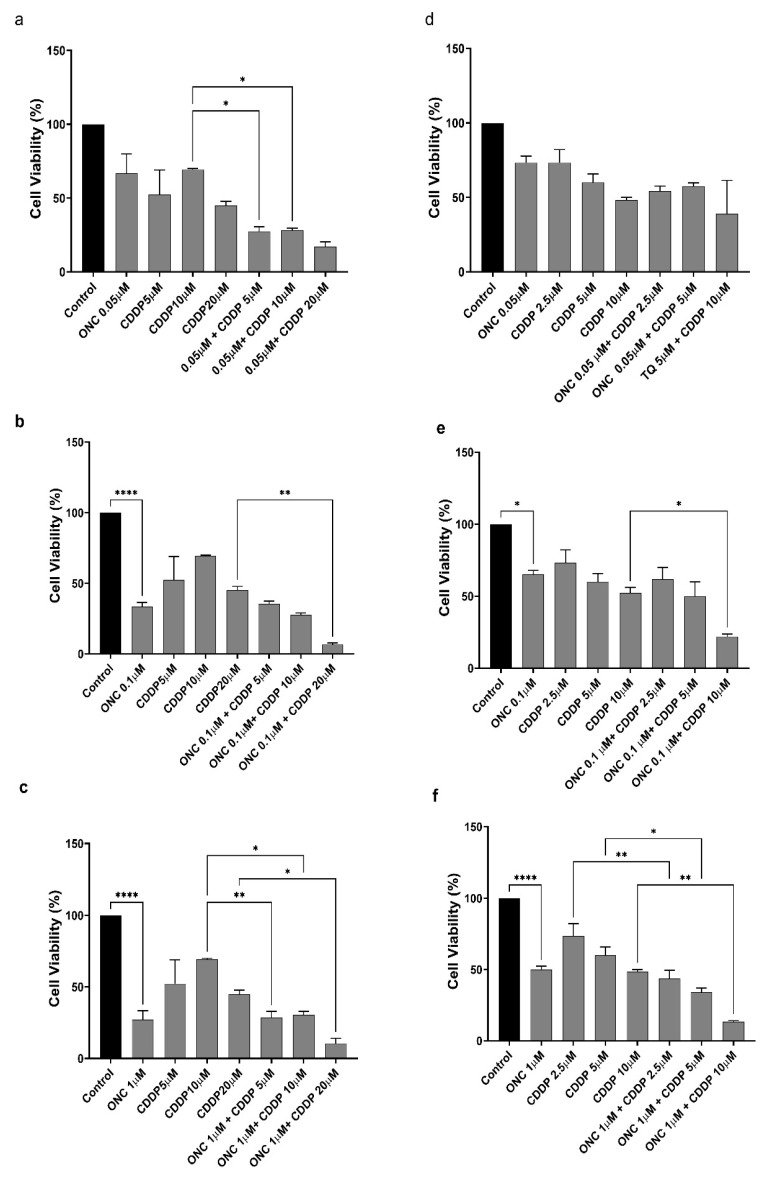
ONC206 and CDDP reduce the viability of OVCAR-420 and SKOV-3 cells. The effect of different concentrations of ONC206 and CDDP alone and in combination on the viability of OVCAR-420 and SKOV-3 cells using trypan blue exclusion assay was determined at 48 h. (**a**–**c**) OVCAR-420 and (**d**–**f**) SKOV-3. Results are expressed as a percentage of the viability of the treated groups compared to the control at every concentration. Data represent the mean ± SEM of three independent experiments and are analyzed using two-way ANOVA (* *p* < 0.05; ** *p* < 0.01; **** *p* < 0.0001).

To assess the effects of CDDP and ONC206, either alone or in combination, on the viability of SKOV-3 and OVCAR-420 cells, a trypan blue exclusion assay was performed (Figure 4). Based on the IC_50_ values (Table 1), SKOV-3 cells were treated with 2.5, 5, or 10 μM of CDDP, whereas OVCAR-420 cells were treated with 5, 10, or 20 μM of CDDP. Both cell lines were also exposed to ONC206 (0.05 μM, 0.1 μM, 1 μM).

In OVCAR-420 cells, ONC206 at 0.1 µM and 1 µM significantly reduced cell viability by approximately 60–70%. Notably, the combination of 0.1 µM ONC206 with 20 µM CDDP further reduced viability by 80–90% relative to the control and induced a statistically significant decrease compared to CDDP alone. Similarly, the combination of 1 µM ONC206 with all tested concentrations of CDDP resulted in a 70–90% reduction in cell viability compared to CDDP alone.

In SKOV-3 cells, ONC206 at 0.1 µM decreased viability by approximately 40%, while a more pronounced reduction of 50% was observed at 1 µM. Notably, co-treatment with 0.1 µM ONC206 and 10 µM CDDP further decreased viability by 70–80% relative to the control and by an additional 30% compared to CDDP alone. Additionally, the combination of 1 µM ONC206 with all tested CDDP concentrations significantly reduced cell viability relative to CDDP alone. These findings were further supported by observable morphological changes. Control groups treated with NaCl or DMSO showed no significant effects on cellular viability (Figure 4).

### 3.5. ONC206 and CDDP Exhibit Synergistic Effects on Ovarian Cancer Cells

To validate the potential synergistic effect of combining ONC206 with CDDP, we evaluated the results from the trypan blue exclusion assay using the CompuSyn tool. This tool calculates the combination index by assessing how the combined effect of the two agents impacts cell viability.

**Figure 5:**
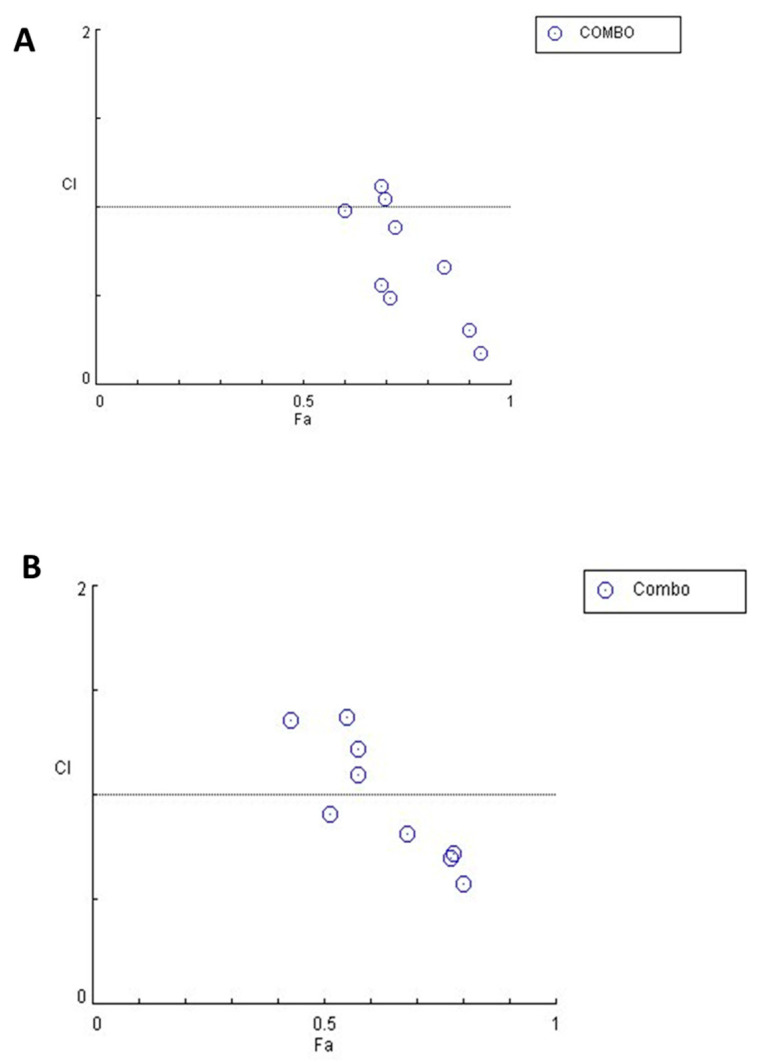
CI-Fa (combination index—Fraction affected) plot of OVCAR-420 and SKOV-3 cells. (**A**): OVCAR-420; (**B**): SKOV-3. The graphs were generated by the computerized software CompuSyn. The circles in the graph indicate the CI values. The synergy analysis shows if the effect of combining two drugs together is greater than their individual effects. (Antagonism CI > 1; Additive CI = 1; Synergism CI < 1).

In OVCAR-420, the combination of ONC206 with CDDP showed a trend of synergism with increasing concentration. This indicated a pattern of higher effects when combining the two drugs together (Table 2; Figure 5A). Similarly, in SKOV- 3 cells, the combinations of ONC206 and CDDP showed a pattern of a synergistic trend with increasing concentrations. This was illustrated graphically (Table 3; Figure 5B).

### 3.6. ONC206 Reduced the Migration of Ovarian Cancer Cell Lines Alone and in Combination with CDDP

To assess the effects of ONC206 and CDDP on the migratory potential of SKOV-3 and OVCAR-420 cell lines, either alone or in combination, we conducted a wound healing assay. Wound areas were measured at 0, 12, and 24 h, with representative time points quantified and presented in the corresponding graphs.

**Figure 6:**
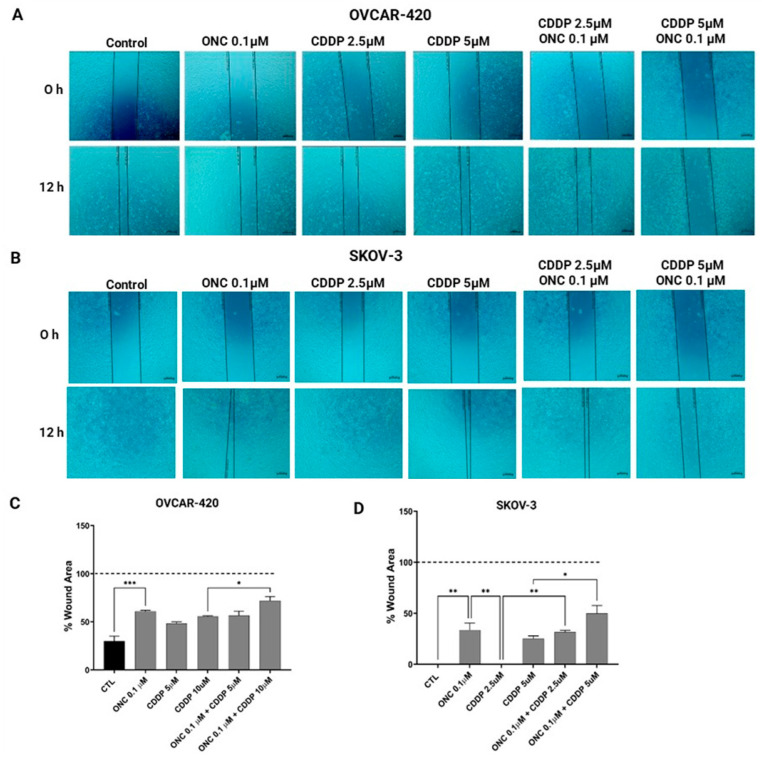
ONC206 reduces the migration of OVCAR-420 and SKOV-3 cells alone and in combination with CDDP. (**A**) OVCAR-420 and (**B**) SKOV-3. The quantification of wound closure was assessed over time by measuring the percentage of the wound area remaining open relative to the initial wound size at 0 h (**C**,**D**). Data represent the mean ± SEM of three independent experiments and are analyzed using two-way ANOVA (* *p* < 0.05; ** *p* < 0.01; *** *p* < 0.001) (light microscope ×10 objective).

At 12 h, untreated OVCAR-420 cells exhibited extensive migration, with approximately 80% of the initial wound area closed (Figure 6A). Treatment with CDDP delayed wound closure in a concentration-dependent manner, resulting in significantly larger remaining wound areas of approximately 50% with 5 µM CDDP and 60% with 10 µM CDDP. Notably, treatment with 0.1 µM ONC206 alone led to a pronounced inhibition of migration, with around 60% of the wound area remaining unclosed. The combination of 0.1 µM ONC206 with 10 µM CDDP further delayed migration, yielding an unclosed wound area of almost 80%, a significant increase compared to 10 µM CDDP alone (Figure 6A,C).

In SKOV-3 cells, both the untreated control and 2.5 µM CDDP-treated conditions achieved complete wound closure by 12 h (Figure 6B,D). In contrast, treatment with either 5 µM CDDP or 0.1 µM ONC206 alone resulted in incomplete closure, with approximately 30–40% of the wound area remaining unhealed, indicating the partial delay of cell migration. Interestingly, the combination of 0.1 µM ONC206 with 2.5 µM CDDP resulted in a persistent wound area of ~40%, suggesting that ONC206 counteracts CDDP-induced closure at lower doses. Similarly, co-treatment with 0.1 µM ONC206 and 5 µM CDDP significantly impaired cell migration, retaining approximately 50% of the initial wound area.

### 3.7. ONC206 Decreases the Growth of Cell-Derived Spheres of Ovarian Cancer Cells Alone and in Combination with CDDP

**Figure 7:**
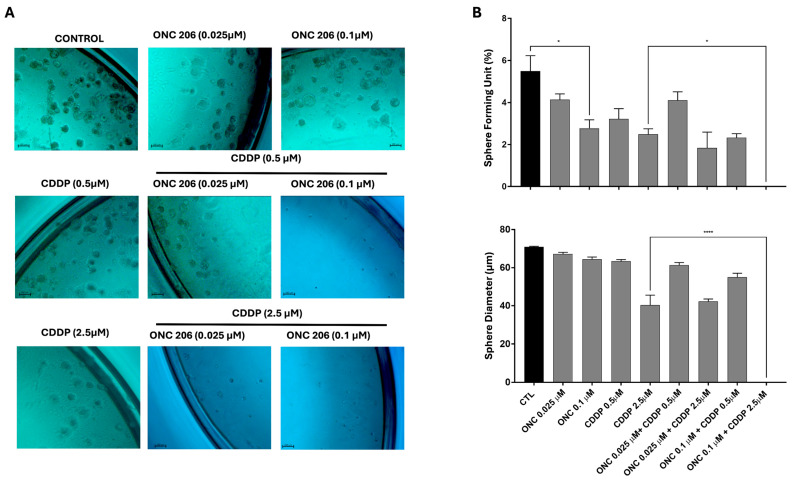
Effect of ONC206 and CDDP, alone or in combination, on the sphere-forming ability and sphere size in OVCAR-420. (**A**) Representative images of spheres in the presence or absence of ONC206 and/or CDDP in OVCAR-420 cells. Images were visualized by inverted light microscope and analyzed by ZEN image software. Scale bar = 100 μm. (**B**) Results are expressed as SFUs (sphere-forming units). Spheres sizes were measured by Carl Zeiss Zen 2012 image software. Data represent an average diameter (μM) of all counted spheres. Data represent the mean ± SEM of three independent experiments and are analyzed using one-way ANOVA (* *p* < 0.05; **** *p* < 0.0001; treatment compared with control) (light microscope ×10 objective).

**Figure 8:**
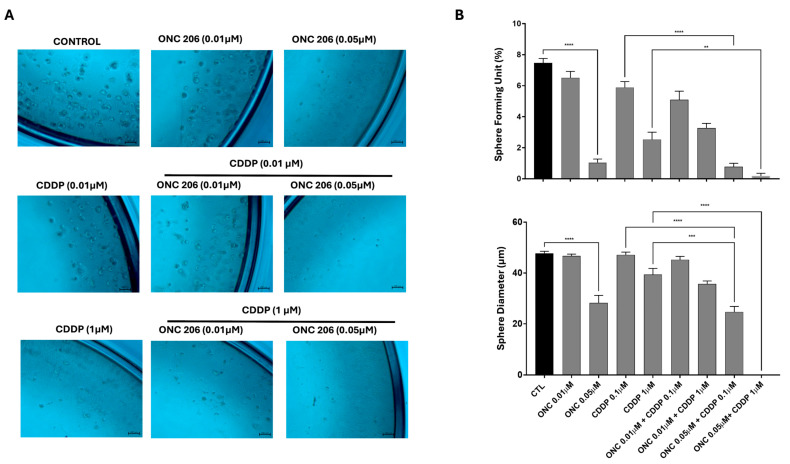
Effect of ONC206 and CDDP, alone or in combination, on the sphere-forming ability and sphere size in SKOV-3 cells. (**A**) Representative images of spheres in the presence or absence of ONC206 and/or CDDP in SKOV-3 cells. Images were visualized by inverted light microscope and analyzed by ZEN image software. Scale bar = 100 μm. (**B**) Results are expressed as SFUs (sphere-forming units). Spheres sizes were measured by Carl Zeiss Zen 2012 image software. Data represent an average diameter (μM) of all counted spheres. Data represent the mean ± SEM of three independent experiments and are analyzed using one-way ANOVA (** *p* < 0.01; *** *p* < 0.001; **** *p* < 0.0001; treatment compared with control) (light microscope ×10 objective).

One of the hallmarks of cancer relapse is the cancer stem cell (CSC) population. In order to assess the self-renewal ability of ovarian CSCs, we used the sphere-formation assay. The potential of ONC206 and CDDP, either alone or in combination, was examined by targeting the progenitor cancer cells or CSCs within the tested OC cell lines. OVCAR-420 (Figure 7) and SKOV-3 (Figure 8) were cultured as single cells in Matrigel™ for 7–10 days and treated with a range of concentrations of CDDP and ONC206 either alone or in combination. The spheres were then envisioned under an inverted light microscope and images were taken. Our data revealed that both cell lines developed spheres, with SKOV-3 cells forming a higher number of sphere-forming units (SFUs) compared to OVCAR-420.

Treatment of OVCAR-420 cells with ONC206 at concentrations of 0.1 µM significantly reduced the number of SFUs (Figure 7). A higher statistically significant reduction was observed when the 0.1 µM of ONC206 was combined with CDDP at 2.5 µM, significantly exceeding the effects of CDDP alone, leading to the complete eradication of the spheres. Interestingly, sphere diameter was significantly reduced only when ONC206 at 0.1 µM was combined with CDDP at 2.5 µM (Figure 7).

Treatment of SKOV-3 cells with ONC206 at concentrations of 0.05 µM significantly reduced the number of SFUs and sphere size (Figure 8). Combining this concentration of ONC206 (0.05 µM) with all tested concentrations of CDDP resulted in a greater significant reduction in both SFUs and sphere diameter relative to CDDP alone.

## 4. Discussion

OC is considered the most lethal gynecological disease worldwide, primarily due to late-stage diagnosis, chemoresistance, and metastasis. Despite advances in surgery, chemotherapy, and molecular-targeted therapies, outcomes remain suboptimal, highlighting the urgent need for innovative therapeutic strategies [23]. Platinum-based agents such as cisplatin (CDDP) are the cornerstone of OC treatment; however, resistance to these agents limits their long-term efficacy. Emerging therapeutics, such as imipridones, offer a promising avenue for overcoming resistance and improving treatment outcomes. ONC206, a second-generation imipridone, has been shown to exhibit potent anti-cancer activity through multiple mechanisms, including the antagonism of dopamine receptor D2 (DRD2), CLpP agonism, and TRAIL activation, disrupting mitochondrial protein homeostasis and inducing cellular stress responses [19,24].

Although ONC206 has been investigated in various types of cancers, including high-grade glioma and endometrial and hepatocellular cancer, its role in OC remains underexplored. Additionally, there are no published studies examining the combination of ONC206 with conventional chemotherapy like cisplatin, leaving a gap in the understanding of its full therapeutic potential. To the best of our knowledge, our study is the first to evaluate ONC206 in combination with CDDP in ovarian cancer cells lines, revealing synergistic anti-tumor effects in both 2D and 3D culture systems.

In our study, we examined the effects of ONC206 alone and in combination with CDDP on two human OC cell lines, SKOV-3 and OVCAR-420, using 2D culture models. Both cell lines were derived from epithelial tissue, with OVCAR 420 originating from HGSC, known for its aggressiveness, and SKOV-3 from a non-serous origin, extracted from metastasized ascites [25,26]. To assess treatment impact, we employed MTT assays, which measure cellular metabolic activity as a proxy for both viability and proliferation. While MTT does not directly quantify proliferation, changes in metabolic activity are widely recognized to correlate with cell number and can reflect proliferative capacity, especially when supported by complementary viability data [27,28].

Our results revealed similar trends across both cell lines, with ONC206 and CDDP reducing cellular proliferation and viability in a dose-dependent manner. Notably, SKOV-3 cells exhibited a threefold higher IC_50_ for ONC206 compared to OVCAR-420, likely due to differences in origin, genetic differences, membrane permeability, or intrinsic metabolic profiles. These results align with those of Tucker et al. [19], who reported a similar IC_50_ value (0.37 μM) for ONC206 in SKOV-3 cells. Moreover, our findings support the growing body of literature highlighting the anti-cancer potential of ONC206. For example, Monzer et al. [29] reported that ONC206 more effectively suppressed proliferation, viability, migration, and invasion than its parent compound, ONC201, in colorectal cancer cell lines. These effects were accompanied by increased ROS generation and cell cycle arrest in both 2D and 3D cell culture models. Similarly, Cao et al. [15] demonstrated that ONC206 effectively suppressed hepatocellular carcinoma (HCC) growth by inhibiting cell proliferation, inducing apoptosis, and disrupting mitochondrial function via ClpP-mediated pathways. Furthermore, ONC206 activated cytoprotective autophagy, and its inhibition significantly amplified proapoptotic effects.

Although ONC206 has demonstrated synergy with other treatments, such as in glioblastoma [30], our study is the first to evaluate its combination with CDDP in OC. The ONC206-CDDP combination significantly reduced cellular viability and proliferation, more than either agent alone, suggesting a synergistic therapeutic interaction. While no previous studies have examined ONC206 in combination with platinum agents in OC, related imipridones, like ONC201, have shown promise. For instance, He et al. [30] reported that ONC201 sensitized glioblastoma cells to radiation by reducing bulk cell viability, impairing the self-renewal capacity of glioma-initiating cells, and suppressing pathways involved in DNA repair and metabolism. These findings support the rationale for exploring ONC206 as a platinum-sensitizing agent in OC.

Cancer cell migration is a critical factor in metastasis, which is partly responsible for the poor prognosis of advanced OC patients [31,32]. Although HGSC is generally more aggressive, the non-serous SKOV-3 cells exhibited higher migratory capabilities compared to OVCAR-420. This finding is supported by previous studies where SKOV-3 has had higher migratory abilities than HGSCs [26,33]. ONC206’s ability to inhibit migration in both cell lines, particularly when combined with CDDP, is a significant finding. This aligns with the study by Zhang et al. [34], which demonstrated that ONC206 significantly impaired cell adhesion and migration in serous endometrial cancer models. The epithelial–mesenchymal transition (EMT), a key process that enhances the migratory and invasive properties of cancer cells, plays a pivotal role in cancer metastasis [35]. The observed reduction in migration could be attributed to interference with signaling pathways regulating EMT and cytoskeletal remodeling [36]. However, further investigation is required to elucidate the specific mechanisms involved and determine whether EMT-related pathways are being targeted by this combination. Cisplatin alone allowed for partial wound closure, but when combined with ONC206, the two drugs demonstrated a synergistic effect, completely inhibiting wound closure. This underscores their potential as a highly effective therapeutic combination, capable of targeting both cancer cell survival and migration.

Most OC patients relapse after initial surgery and chemotherapy, reflecting a subpopulation of OCSCs with a self-renewal capacity. The subpopulation is believed to be a key driver of tumor initiation, progression, cancer relapse, and conventional therapy resistance [12,37]. OCSCs have been shown to exhibit properties such as enhanced tumorigenicity and the ability to initiate tumor growth, which contributes to the challenges associated with effective treatment [38]. In this study, both OVCAR-420 and SKOV-3 cell lines demonstrated a sphere-forming ability in 3D cultures, consistent with the presence of a stem-like population. Treatment with ONC206, especially in combination with CDDP, significantly reduced SFUs in both cell lines. Notably, SKOV-3 cells, which initially showed higher SFU numbers, exhibited a more pronounced reduction in sphere diameter, suggesting a greater sensitivity in this morphological context. This response may reflect heterogeneity in cancer stem cell subpopulations or different cellular mechanisms regulating sphere formation and growth [39]. Therefore, future mechanistic studies should investigate the basis of the differences.

While previous studies have highlighted the ability of ONC206 to target CSC populations in colorectal models [29], our study is the first to demonstrate this effect in OC. Previous studies in fresh ovarian carcinoma samples have highlighted that OCSCs express specific markers, such as CD44, CD133, and aldehyde dehydrogenase (ALDH) activity, which are associated with stemness and may play a role in therapeutic resistance [40]. Thus, although ONC206 reduced the ability of cells to form spheres—a functional hallmark of stem-like behavior—further validating using molecular CSC markers is warranted. Our study underscores the need for further investigation to understand the behavior of OC in less aggressive yet metastasized regions, as elucidating the mechanisms underlying OCSC survival and resistance could lead to more effective therapeutic strategies targeting this elusive population.

## 5. Conclusions

Our study is the first to demonstrate that the combination of cisplatin (CDDP) with ONC206 exhibits synergistic anti-cancer effects in ovarian cancer (OC), achieving greater efficacy than CDDP alone. Our data confirm the efficacy of ONC206 at nanomolar concentrations in both 2D and 3D culture systems, underscoring its potential as both a monotherapy and in combination with CDDP for OC treatment. These results position ONC206 as a promising candidate for future OC clinical trials. To further substantiate these findings, future research should evaluate the effects of ONC206 and its combination with CDDP across a broader range of OC cell lines, representing various stages and subtypes of the disease. Conducting additional bio-functional assays, including trans-well invasion assays, cell cycle analysis, and reactive oxygen species (ROS) production assays, will be essential for delineating the drugs’ impact on diverse cancer hallmarks. Moreover, it will be important to investigate the underlying mechanisms of cell death induced by ONC206 and CDDP, including the evaluation of both apoptotic (e.g., cleaved PARP, caspase-3, BAX, BAK, BCL-2, BCL-XL) and potential non-apoptotic markers to gain a comprehensive understanding of the pathways involved. In vivo studies are also crucial to validate the in vitro outcomes and to provide a comprehensive understanding of ONC206’s therapeutic potential in a more complex biological context. Additionally, evaluating ONC206 in 3D patient-derived ovarian cancer organoid models could offer a more physiologically relevant pre-clinical platform that closely replicates the genetic, histological, and physiological features of patient tumors. These comprehensive investigations will be pivotal in translating our findings into clinical settings and advancing therapeutic strategies for OC.

## Figures and Tables

**Table 1:** Half-maximal concentration values.

Cell Lines	OVCAR-420			SKOV-3		
Time points	24 h	48 h	72 h	24 h	48 h	72 h
IC_50_ of CDDP	16.85 μM	10.92 μM	8.62 μM	6.8 μM	5.3 μM	5.2 μM
IC_50_ of ONC206	0.16 μM	0.09 μM	0.152 μM	0.336 μM	0.27 μM	0.245 μM

**Table 2:** Combination index (CI) and the relative indication in OVCAR-420 cells.

Dose CDDP	Dose ONC206	Effect	CI	Indication
5.0	0.05	0.69	0.55963	Synergism
10.0	0.05	0.7	1.04693	Additive
20.0	0.05	0.84	0.66549	Synergism
5.0	0.1	0.6	0.97897	Synergism
10.0	0.1	0.72	0.88727	Synergism
20.0	0.1	0.93	0.17915	Synergism
5.0	1.0	0.71	0.49044	Synergism
10.0	1.0	0.69	1.12129	Additive
20.0	1.0	0.9	0.31066	Synergism

**Table 3:** Combination index (CI) and the relative indication in SKOV-3 cells.

Dose CDDP	Dose ONC206	Effect	CI	Indication
2.5	0.05	0.43	1.35872	Antagonism
5.0	0.05	0.55	1.37010	Antagonism
10.0	0.05	0.775	0.70073	Synergism
2.5	0.1	0.5125	0.90821	Synergism
5.0	0.1	0.575	1.22318	Antagonism
10.0	0.1	0.8	0.57864	Synergism
2.5	1.0	0.575	1.09485	Additive
5.0	1.0	0.68	0.81825	Synergism
10.0	1.0	0.78	0.71879	Synergism

## Data Availability

The original contributions presented in this study are included in the article. Further inquiries can be directed to the corresponding author.
